# Sleep health differences and night-to-night variability in people with and without HIV in Mwanza, Tanzania: insights from actigraphy and sleep diaries

**DOI:** 10.1093/sleepadvances/zpag092

**Published:** 2026-08-18

**Authors:** Godfrey A Kisigo, Martha Oshosen, Vanessa Nyamkomola, Ezekiel Mgema, Megan Willkens, Shelley Lees, Robert N Peck, Ana C Krieger, Saidi Kapiga, Kathy Baisley

**Affiliations:** Department of Infectious Disease Epidemiology, London School of Hygiene and Tropical Medicine, London, United Kingdom; Mwanza Intervention Trials Unit, National Institute for Medical Research, Mwanza, Tanzania; Department of Non-Communicable Diseases, Kilimanjaro Clinical Research Institute, Moshi, Tanzania; Department of Non-Communicable Diseases, Kilimanjaro Clinical Research Institute, Moshi, Tanzania; Mwanza Intervention Trials Unit, National Institute for Medical Research, Mwanza, Tanzania; Center for Global Health, Department of Medicine, Weill Cornell Medicine, New York, New York, United States; Department of Infectious Disease Epidemiology, London School of Hygiene and Tropical Medicine, London, United Kingdom; Mwanza Intervention Trials Unit, National Institute for Medical Research, Mwanza, Tanzania; Center for Global Health, Department of Medicine, Weill Cornell Medicine, New York, New York, United States; Department of Medicine, Weill Bugando School of Medicine, Mwanza, Tanzania; Weill Cornell Medicine Center for Sleep Medicine, Department of Medicine, Neurology and Genetic Medicine, Weill Cornell Medicine, New York, New York, United States; Department of Infectious Disease Epidemiology, London School of Hygiene and Tropical Medicine, London, United Kingdom; Mwanza Intervention Trials Unit, National Institute for Medical Research, Mwanza, Tanzania; Department of Infectious Disease Epidemiology, London School of Hygiene and Tropical Medicine, London, United Kingdom

**Keywords:** HIV, sleep variability, epidemiology, actigraphy, Mwanza HIV & CVD cohort, Tanzania

## Abstract

**Study Objectives:**

This study had two objectives: (1) to compare objectively and subjectively measured sleep health between people living with HIV (PLWH) and people without HIV (PWoH) in Mwanza, Tanzania, and (2) to determine the number of nights of actigraphy and sleep diary monitoring required to obtain reliable estimates of habitual sleep.

**Materials and Methods:**

We conducted a cross-sectional study among participants enrolled in the Mwanza HIV and CVD Cohort. Participants completed seven consecutive nights of wrist actigraphy and daily sleep diaries. Sleep health parameters were compared between PLWH and PWoH using linear mixed-effects models for repeated measures and linear regression for participant-level variability measures**.** Intraclass correlation coefficients and the Spearman–Brown formula were used to estimate the number of nights of monitoring required for reliable measurement.

**Results:**

Of the 100 participants (50 PLWH and 50 PWoH), 70% were female, and the median age was 49 years (IQR 44–54). Based on actigraphy data, PLWH had shorter mean sleep duration than PWoH (adjusted mean difference (aMD) −32.1 minutes, 95% confidence interval (CI) −52.6, −11.7). Bedtimes were slightly later (aMD 17.4 minutes, 95% CI −2.4, 37.3) and wake-up times slightly earlier (aMD −2.1 minutes, 95% CI −21.6, 17.3) among PLWH compared with PWoH. PLWH also exhibited greater night-to-night variability in sleep duration (aMD in standard deviation 13.1 minutes, 95% CI 1.2, 25.0). Actigraphy-derived sleep measures required at least 7 nights for reliable estimates, whereas self-reported sleep quality required at least 3 nights.

**Conclusions:**

These findings highlight the importance of multi-day actigraphy for accurately characterizing sleep in sub-Saharan Africa and suggest that HIV is associated with shorter, less consistent sleep without altering circadian timing.

## Graphical Abstract

**Graphical Abstract:**
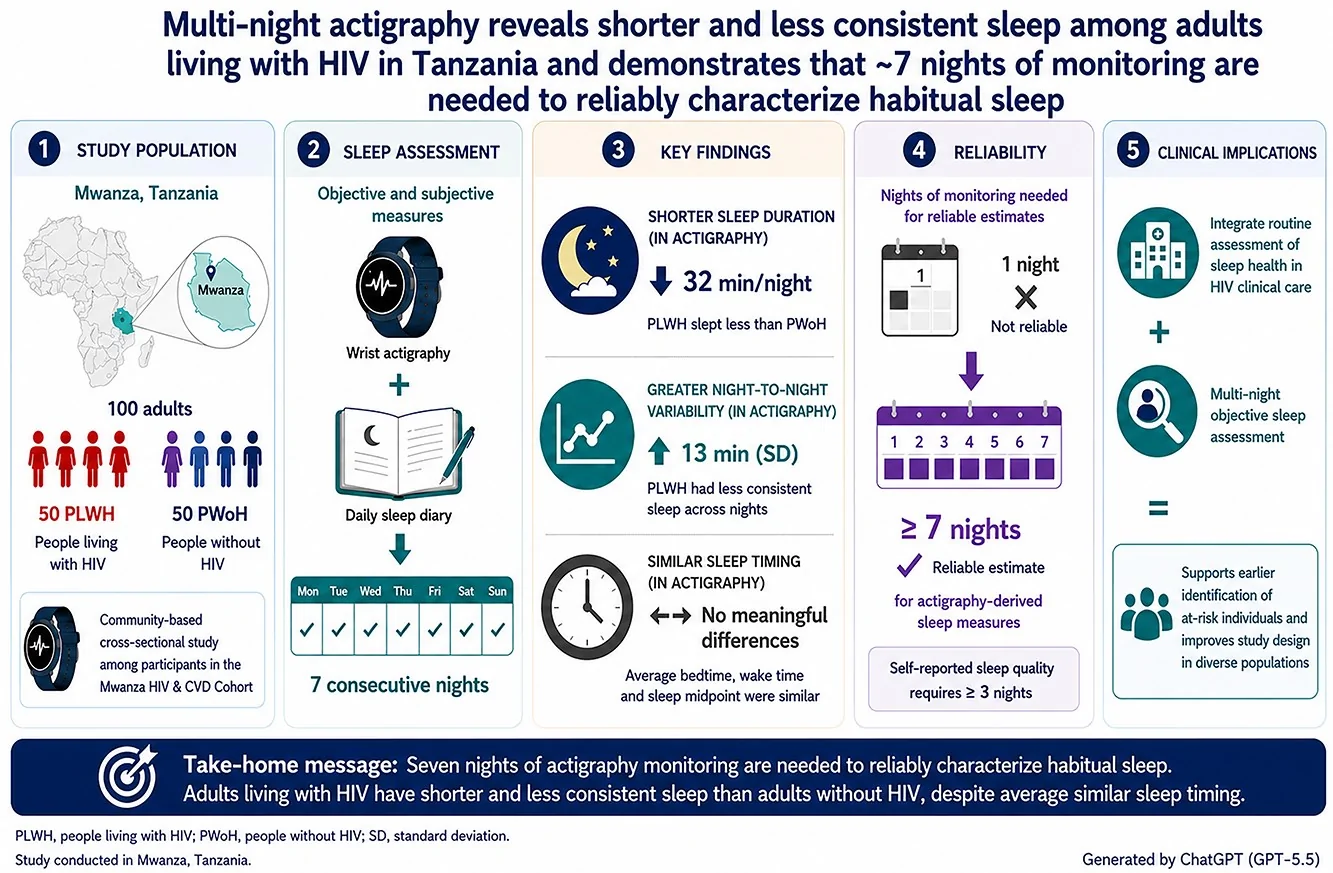

**Statement of Significance**

This study provides novel evidence on sleep patterns and night-to-night variability in a Sub-Saharan African population using multi-night actigraphy and sleep diaries. In a well-characterized cohort, we show that people living with HIV have shorter sleep duration and greater night-to-night variability compared to those without HIV, despite similar sleep timing. Importantly, the study demonstrates that at least seven nights of actigraphy are required to obtain reliable estimates of sleep duration, highlighting the limitations of short monitoring periods. By integrating objective and subjective measures, this work advances understanding of sleep by highlighting the importance of accounting for night-to-night variability. It addresses a critical gap in global sleep epidemiology, with direct implications for study design and sleep assessment in diverse populations.

## Introduction

Sleep health is increasingly recognized as a determinant of physical and mental well-being, playing an important role in maintaining systemic physiological function [1]. Growing evidence links insufficient or irregular sleep to adverse outcomes, including cardiovascular disease, metabolic disorders, and psychological distress [2, 3]. Some studies have reported poor sleep health among people living with HIV (PLWH) [4–6]. Sleep abnormalities in PLWH may be driven by HIV-induced chronic inflammation [7], the neurotropic side effects of certain antiretroviral therapies (ART) [8, 9], and the persistent psychosocial and socioeconomic stressors associated with HIV including stigma [10].

In sub-Saharan Africa (SSA), studies examining the burden and health implications of poor sleep are limited. Furthermore, most studies conducted in SSA have used self-reported questionnaires [11, 12], which are prone to recall bias and overestimate sleep quantity compared to objective measures [13]. In addition, retrospective sleep questionnaires are limited in their ability to capture night-to-night variability in sleep patterns, an increasingly recognized dimension of sleep health [3, 14, 15]. Consequently, studies using objective measures over multiple nights are needed in SSA to more accurately characterize sleep health, including sleep regularity and variability, and to determine the minimum monitoring duration needed to obtain reliable estimates of sleep patterns.

Among objective methods of assessing sleep, actigraphy is widely used in epidemiological studies because it allows monitoring sleep in a natural environment for extended periods [16]. Compared with polysomnography (PSG), a gold standard, actigraphy is less expensive and easier to use in large population-based studies [17]. Our group recently reported baseline sleep health findings from the Mwanza HIV & CVD Cohort using a single-night objective assessment alongside retrospective questionnaires, demonstrating modest yet important differences in sleep health between PLWH and people without HIV (PWoH) [18]. However, that study could not characterize within-person night-to-night variability or determine the monitoring duration required to obtain reliable estimates of habitual sleep. Building on that work, this nested substudy was designed to further examine sleep variability and evaluate the reliability of repeated sleep measurements during seven consecutive nights of wrist actigraphy and daily sleep diaries. Specifically, this study had two complementary objectives: (1) to compare objectively and subjectively measured sleep health between PLWH and PWoH, including sleep duration, timing, quality, and night-to-night variability, and (2) to determine the minimum number of monitoring nights required to obtain reliable estimates of habitual sleep.

## Materials and methods

### Overview

This cross-sectional sleep substudy was nested within the ongoing Mwanza HIV&CVD Cohort, a prospective cohort study of 500 PLWH and 500 PWoH established to investigate the interrelationships between HIV infection, sleep, and cardiovascular diseases (CVD) in Mwanza, Tanzania [19–22]. Participants in the parent cohort underwent comprehensive baseline assessments and were followed prospectively for 36 months. The present substudy was conducted during the endline (36-month) follow-up visit, when participants completed seven consecutive nights of wrist actigraphy and daily sleep diaries to provide a more detailed assessment of habitual sleep. The study protocol was approved by the Tanzanian National Institute for Medical Research [Ref: NIMR/HQ/R.8a/Vol. IX/4713], London School of Hygiene and Tropical Medicine [Ref: 30550], and Weill Cornell Medicine [Ref: 23-06027628-02]. Study methods and results are reported following the Strengthening the Reporting of Observational Studies in Epidemiology Statement for cross-sectional studies [24].

### Study participants

Participants were consecutively recruited from individuals attending the scheduled 36-month (endline) follow-up visit of the parent cohort at the study clinic between April and July 2025. Each day, all 8 participants completing the endline visit during the recruitment period were invited to learn more about the substudy until a target of three participants had been recruited (to match the number of actigraphy devices available). The target sample size of 100 participants was not based on a formal power calculation but reflected a pragmatic balance between device availability, the logistical demands of seven consecutive nights of monitoring, and the objective of obtaining high-quality repeated sleep measurements. The research assistant described the study procedures to prospective participants, and those who expressed interest were screened for eligibility and provided written informed consent. Participants were eligible if they were willing to wear a wrist actigraphy for 7 days and complete daily sleep diaries. Night shift workers were excluded. Inclusion and exclusion criteria of the parent Mwanza HIV&CVD Cohort are described elsewhere [22]. We monitored recruitment to ensure balanced enrollment by HIV status, aiming to recruit 50 PLWH and 50 PWoH, with 35 women in each group to approximate the sex distribution of the parent cohort, thereby supporting balanced between-group comparisons while maintaining the parent cohort’s representativeness.

### Sleep measurements

#### Sleep diary

Subjective sleep was assessed using the Karolinska Sleep Diary (KSD) [23, 25], a validated daily sleep diary designed to measure night-to-night variations in self-reported sleep. Participants completed the diary each morning to report sleep characteristics from the preceding night during the 7 consecutive days of monitoring. The diary captured sleep timing measures, including time to bed, lights-off time, sleep onset latency, and wake-up time, as well as 7 items to assess subjective sleep quality. A sleep quality index (SQI), as originally described by Åkerstedt et al. [23], was calculated using the four KSD items: (1) sleep quality phrased “How did you sleep?” (very well [5]—very poorly [1]); (2) restless sleep (not at all [5]—very restless [1]); (3) difficulty falling asleep (not at all [5]—very difficult [1]); and (4) premature (final) awakening (not at all [5]—woke up far too early [1]). Responses were recorded on a five-point Likert scale and summed up to produce a total score ranging from 4 to 20, with higher scores indicating better perceived sleep quality.

#### Actigraphy

Objective sleep parameters were assessed using a wrist-worn actigraph, Micro Motionlogger Watch (Micro Motionlogger®, Ambulatory Monitoring, Inc., Ardsley, New York, USA). The actigraph contains a solid-state triaxial accelerometer that measures acceleration along three orthogonal axes (*x, y*, and *z*) to capture wrist movement and estimate sleep–wake patterns based on periods of activity and inactivity. Actigraphy-derived sleep estimates from the Motionlogger show good agreement with PSG-scored sleep in adults [17]. Participants were requested to wear the actigraph on their non-dominant wrist continuously for the 7 consecutive days of monitoring, except during activities that could damage the device (e.g. prolonged water exposure). The first day (night) of measurement was either a Wednesday (*n* = 35), Thursday (*n* = 38), or Friday (*n* = 27).

Activity data were recorded in 60-second epochs and sleep–wake scoring was performed using the Cole–Kripke algorithm implemented in Action-W (AW2) software (version 2.7). Bedtime and wake-up times were determined using participants’ self-reports from the sleep diary and verified through visual inspection of activity plots. When discrepancies were observed, the actigraphy activity pattern was visually inspected, and the rest interval was adjusted accordingly [17].

Each actigraphy recording underwent manual quality-control review in Action-W before sleep scoring. Suspected non-wear periods were identified through visual inspection of synchronized activity, Life Measures, and case temperature channels. Off-wrist periods were defined as a sustained reduction or absence of the proprietary Life Measures signal, accompanied by a rapid decline in case temperature, and bounded by activity patterns consistent with device removal and reapplication. Identified non-wear intervals were marked as “BAD” in Action-W prior to analysis, in accordance with the manufacturer’s recommended quality-control procedures and consistent with previous methodological applications of wrist actigraphy [16, 17]. A valid night required at least 4 hours of continuous, high-quality data between bedtime and wake-up time. Participants were included in the analysis if they had at least three valid weekday nights and at least one valid weekend night of actigraphy data.

Actigraphy-derived sleep variables included sleep duration, sleep onset latency, wake after sleep onset (WASO), and sleep midpoint, defined as the midpoint between sleep onset and final awakening. Night-to-night variability in sleep duration and timing was assessed using the standard deviation of nocturnal sleep duration and the standard deviation of sleep midpoint across the monitoring period. Sleep parameter reports were generated using Action-W (AW2) software.

#### Demographic, behavioral, and clinical data

Participants completed a standardized questionnaire and underwent a physical examination by trained staff at enrollment. The questionnaire collected demographic characteristics including age, sex, education level, and employment status, as well as behavioral factors such as alcohol use. Depressive symptoms were assessed using the Patient Health Questionnaire-9 (PHQ-9) [26], a validated nine-item instrument that measures the frequency of depressive symptoms over the preceding two weeks, with total scores ranging from 0 to 27 and higher scores indicating greater depressive symptom severity. Psychological distress was measured using the Kessler-6 Distress Scale (K6) [27], a six-item scale assessing nonspecific psychological distress over the past 30 days, with total scores ranging from 0 to 24 and higher scores indicating greater distress. Sleep hygiene behaviors were assessed using the Sleep Hygiene Index (SHI) [28], a 13-item self-report instrument that evaluates behaviors and environmental factors that may interfere with sleep; total scores range from 13 to 65, with higher scores indicating poorer sleep hygiene. The standardized physical examination, including weight and height measurements, was performed according to the World Health Organization’s (WHO) STEPwise Surveillance protocol [29]. For PLWH, CD4+ T-cell count was measured using an automated BD FACSCalibur System (BD Biosciences). HIV viral loads were extracted from medical records. HIV viral load suppression was defined by an HIV RNA level of less than 1000 copies/mL [30].

### Statistical analysis

Analyses were conducted in Stata 18 (StataCorp LLC, College Station, TX). Baseline demographic, behavioral, and clinical characteristics were summarized by median and interquartile range (IQR) for continuous variables and frequency and percentages for categorical variables.

We compared sleep health parameters between PLWH and PWoH using linear mixed-effects models for outcomes with repeated nightly measurements and standard linear regression models for participant-level measures of variability (the standard deviation of sleep duration and the standard deviation of sleep midpoint), for which a single value per participant was available. Potential confounders were specified a priori based on known risk factors for sleep health that were not thought to be on the causal pathway between HIV status and the outcome. These were age, sex, body mass index (BMI), education level, socioeconomic status, alcohol use, and depressive symptoms.

Reliability of nightly sleep measurements was assessed using intraclass correlation coefficients (ICC) estimated from a mixed effects regression model with fixed effects for night and a random effect for participant [31]. The ICC represents the proportion of total variance attributable to between-person differences relative to within-person (night-to-night) variability and therefore reflects the reliability of a single night of measurement.

The number of monitoring nights required to achieve reliable estimates of sleep parameters was estimated using the Spearman–Brown prophecy formula applied to the ICC. The Spearman–Brown reliability coefficient was calculated as:


$$R_{SB}=\frac{N\times ICC}{1+\left(N-1\right)\times ICC}$$

where *N* represents the number of nights of measurement and the ICC is obtained from the mixed effects model. Reliability coefficients were calculated for each sleep parameter derived from actigraphy (sleep duration, sleep onset latency, and WASO) and for the SQI derived from the sleep diary. A reliability coefficient ≥ 0.70 was considered to indicate acceptable reliability [32].

To examine whether reliability differed between weekday and weekend sleep patterns, ICCs were estimated separately for weekday and weekend nights for each sleep parameter, and the corresponding number of nights required to achieve acceptable reliability was derived using the Spearman–Brown formula.

## Results

### Description of participants

Between April 02 and July 01 2025, 258 participants attended their endline visit in the Mwanza HIV & CVD Cohort. Of these, 116 (45%) participants were screened for eligibility for this substudy. The remaining 142 (55%) participants were not screened because the maximum number of actigraphy devices available for deployment on a given day was reached (*n* = 108; 76%) or because, when offered information about the substudy, they reported not having sufficient time after completing the 36-month visit procedures to receive further information (*n* = 34; 24%). Among the 116 participants screened, 100 (86%) met the eligibility criteria and were enrolled in the study. The remaining 16 (14%) participants were not enrolled due to planned travel (*n* = 11; 69%), deliberation regarding participation (*n* = 2; 12%), pre-existing injuries (*n* = 2; 12%), and inability to provide informed consent due to intoxication (*n* = 1, 7%).

Of the 100 participants (50 PLWH and 50 PWoH) enrolled, the median age [IQR] was similar between the two groups (49 [44-56] and 48 [45-52] years, respectively). Overweight and obesity, defined according to WHO criteria (BMI ≥25 kg/m^2^), were more common among PWoH (58%) than among PLWH (34%). Among PLWH, the median CD4 cell count was 680 cells/µL, and 96% (44 out of 46 with HIV viral load results) were virally suppressed. The majority of PLWH (*n* = 49) were on an integrase inhibitor–based antiretroviral therapy (ART) regimen containing dolutegravir, while one participant was on a protease inhibitor–based regimen containing atazanavir. The detailed demographic characteristics of the study participants are presented in Table 1. The mean SHI score was similar between PLWH and PWoH (23.5 vs 24.0).

**Table 1:** Participants characteristics

Variable	Total (*N* = 100)	PLWH (*n* = 50)	PWoH (*n* = 50)
Sex			
Female	70 (70%)	35 (70%)	35 (70%)
Age in years (median, IQR)	49 (45-54)	49 (44-56)	48 (45-52)
Education level			
No/incomplete primary	15 (15%)	9 (18%)	6 (12%)
Primary school education	73 (73%)	36 (72%)	37 (74%)
Secondary /college/University	12 (12%)	5 (10%)	7 (14%)
Marital status			
Married/cohabiting	56 (56%)	23 (46%)	33 (66%)
Divorced/ widowed/single	44 (44%)	27 (54%)	17 (34%)
Socioeconomic status score^*^ (median, IQR)	3 (2 – 4)	2 (1 – 4)	4 (2 – 4)
Score 0	23 (23%)	15 (30%)	8 (16%)
Score 1	17 (17%)	12 (24%)	5 (10%)
Score 2	18 (18%)	7 (14%)	11 (22%)
Score 3	23 (23%)	8 (16%)	15 (30%)
Score ≥ 4	19 (19%)	8 (16%)	11 (22%)
Current alcohol use			
Yes	22 (22%)	12 (24%)	10 (20%)
BMI (kg/m^2^) (median, IQR)	23.7 (20.5–29.3)	22.7 (20.3–27.2)	25.6 (20.8–30.0)
Underweight <18.5	6 (6%)	3 (6%)	3 (6%)
Normal 18.5–24.9	48 (48%)	30 (60%)	18 (36%)
Overweight 25.0–29.9	26 (26%)	10 (20%)	16 (32%)
Obese ≥30	20 (20%)	7 (14%)	13 (26%)
CD4 count (cells/µL)		680 (434-879)	
Probable depression^†^			
Present	18 (18%)	8 (16%)	10 (20%)
Non-specific serious mental illness^‡^			
Present	7 (7%)	2 (4%)	5 (10%)
SHI score (median, IQR)^§^	24.0 (20.0–28.0)	23.5 (20.0–28.0)	24.0 (20.0–27.0)

PLWH: People Living with HIV, PWoH: People without HIV.

^*^A composite score combining ownership of four household assets (television, refrigerator, motorcycle, and car) and presence of tap water and electricity, each contributing a 1-point.

^†^Patient Health Questionnaire (PHQ-9) score of ≥10.

^‡^Kessler-6 Distress Scale (KDS), a six-item measure of non-specific psychological distress over the past 30 days; items are rated on a 5-point scale (0–4), yielding total scores from 0 to 24, with score of ≥13 indicating serious psychological distress.

^§^SHI, a 13-item self-report measure of sleep hygiene behaviors; items are rated from 1 (never) to 5 (always) and summed to produce total scores ranging from 13 to 65, with higher scores reflecting poorer sleep hygiene.

### Sleep diary–derived sleep quality by HIV status

**Figure 1:**
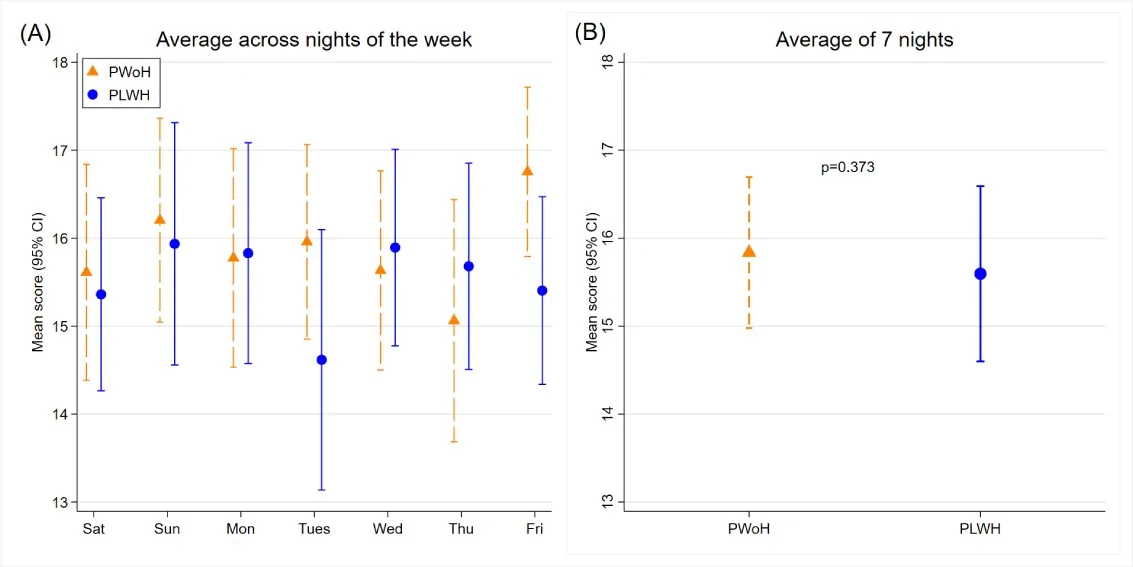
Sleep diary–derived sleep quality index (SQI) across nights of the week (A) and average of 7 nights (B), stratified by HIV status (points indicate means; vertical bars represent 95% confidence intervals).

The group mean values and 95% confidence intervals for SQI by night of the week are presented in Figure 1A. When averaged across the monitoring period (Figure 1B), SQI was slightly lower among PLWH compared to PWoH (mean difference − 0.33, 95% CI −1.62, 0.97). This difference remained non-significant after adjustment for age, sex, BMI, education level, socioeconomic status, alcohol use, and depressive symptoms (*p* = .373).

### Actigraphy-derived sleep health parameters by HIV status

All 100 enrolled participants contributed a total of 691 nights of actigraphy data, with a mean (SD) of 6.9 (0.6) nights per participant. Four participants were excluded from the analysis because their data did not meet the predefined inclusion criteria. Consequently, 96 (47 PLWH and 49 PWoH) participants were included in the analyses, each contributing 7 valid nights, for a total of 672 nights of actigraphy recordings.

**Figure 2:**
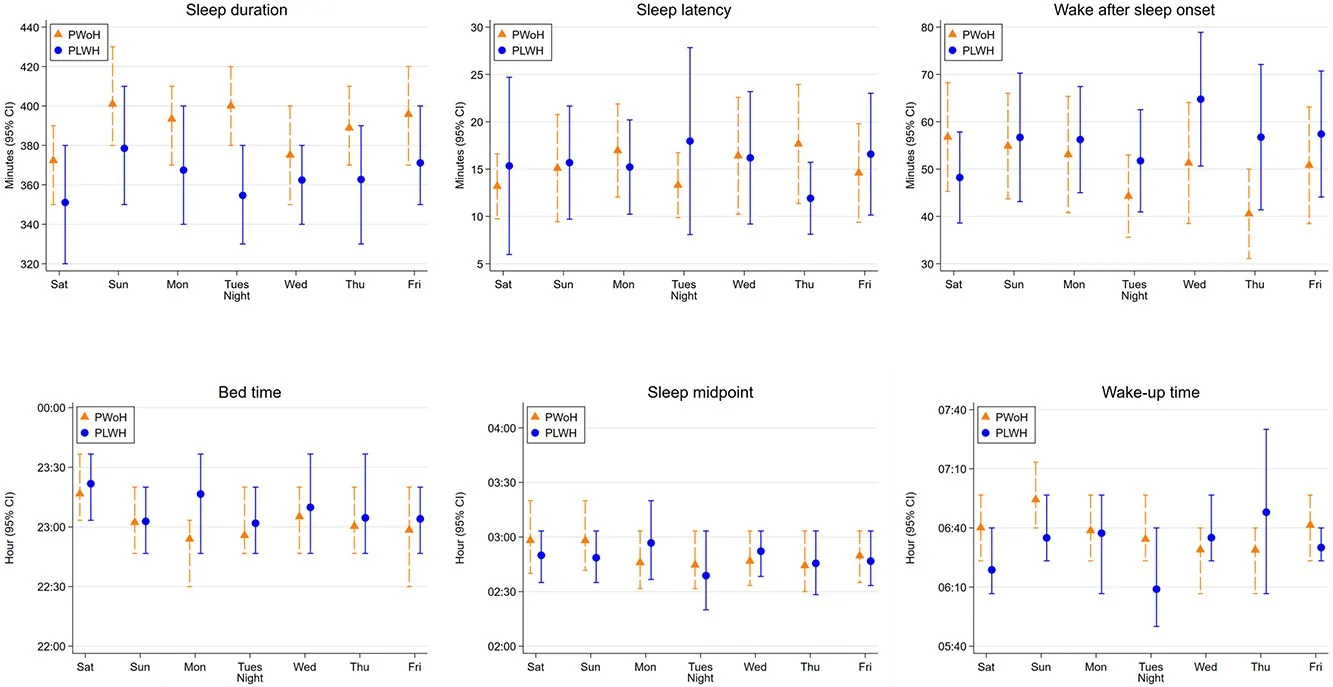
Actigraphy-derived sleep health parameters across nights of the week, stratified by HIV status (points indicate means; vertical bars represent 95% confidence intervals).

Figure 2 shows actigraphy-derived sleep parameters across nights of the week stratified by HIV status. In linear mixed-effects models accounting for repeated nightly measurements, sleep duration was significantly shorter among PLWH compared to PWoH (mean difference −25.5 minutes, 95% CI −46.0, −5.0). There was no statistically significant difference in other sleep parameters between PLWH and PWoH.

Table 2 presents the unadjusted and adjusted mean differences in actigraphy-derived sleep parameters between PLWH and PWoH. PLWH had a mean sleep duration 32 minutes shorter than PWoH (adjusted mean difference −32.1 minutes, 95% CI −52.6, −11.7). Measures of sleep regularity also differed between groups, with PLWH exhibiting greater standard deviation in nocturnal sleep duration compared with PWoH (adjusted mean difference 13.1 minutes, 95% CI 1.2, 25.0).

**Table 2:** Summary of actigraphy-derived metrics by HIV status

	PLWH (*n* = 47) Mean (SD)	PWoH (*n* = 49) Mean (SD)	Mean difference between PLWH and PWoH (95% CI)	Adjusted mean difference between PLWH and PWoH (95% CI)^*^
**Sleep consolidation**				
Mean nocturnal sleep duration (min)	364.0 (55.9)	389.5 (47.5)	**–25.5 (–46.0, –5.0)**	**–32.1 (–52.6, –11.7)**
Mean sleep latency (min)	15.6 (9.8)	15.3 (7.4)	0.2 (–3.2, 3.7)	–0.9 (–4.3, 2.5)
Mean wake after sleep onset (min)	56.0 (27.3)	50.2 (26.6)	5.7 (–4.9, 16.4)	6.3 (–4.4, 17.1)
**Timing of rhythms**				
Bed time	23:08 (0:51)	23:01 (0:48)	7.0 min (–12.8, 26.7)	17.4 min (–2.4, 37.3)
Sleep midpoint	02:47 (0:39)	02:49 (0:40)	–2.5 min (–18.5, 13.4)	4.4 min (–11.8, 20.6)
Wake-up time	06:26 (0:43)	06:37 (0:44)	–7.6 min (–26.6, 11.3)	-2.1 min (–21.6, 17.3)
**Regularity of rhythms**				
Standard deviation of nocturnal sleep duration (min)^†^	74.6 (31.2)	62.7 (24.0)	**11.9 (0.6, 23.2)**	**13.1 (1.2, 25.0)**
Standard deviation of sleep midpoint (min)^‡^	39.0 (20.0)	32.9 (18.6)	6.1 (−1.3, 13.6)	6.5 (–1.4, 14.4)

^*^Adjusted for age, sex, BMI, education level, socioeconomic status, alcohol use, and PHQ scores.

^†^Sleep duration standard deviation represents the within-person standard deviation of nocturnal sleep duration across monitoring nights; higher values indicate less regular sleep rhythms.

^‡^Sleep midpoint SD represents the within-person standard deviation of nightly sleep midpoint; higher values indicate less regular sleep–wake timing.

### Number of nights needed to obtain a reliable sleep measure

**Figure 3:**
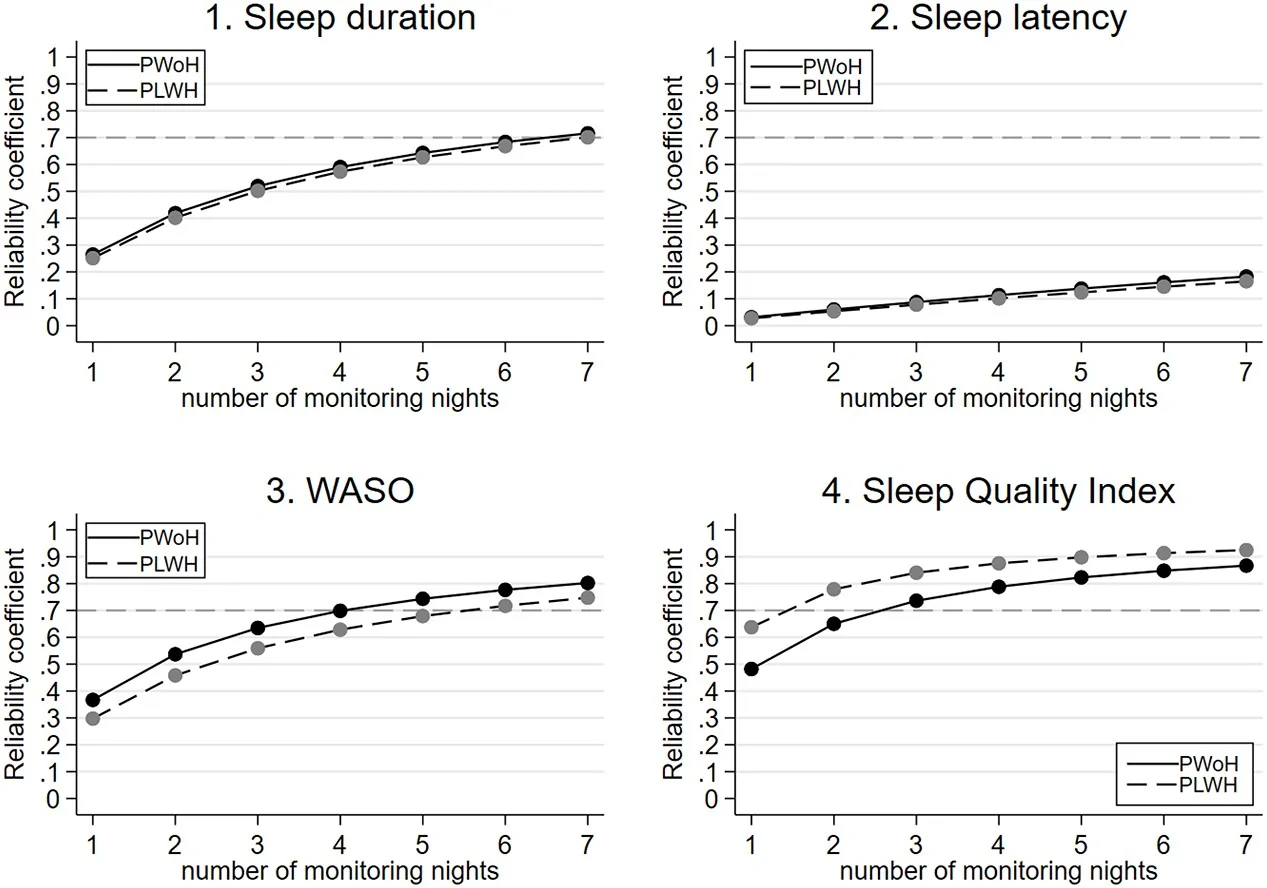
Graph 1–3 present sleep parameters from the actigraphy measures and graph 4 the subjectively reported sleep quality (by index from the sleep diary). Spearman-Brown coefficients >0.70 indicate acceptable reliability. The Spearman-Brown coefficient for 1 night is equivalent to the intraclass correlation coefficient (ICC).

Figure 3 shows reliability coefficients for 1–7 nights of monitoring for each sleep consolidation parameter and self-report sleep quality stratified by HIV status. At least 7 nights of monitoring were required to achieve acceptable reliability for sleep duration in both PLWH and PWoH. Sleep latency did not achieve acceptable reliability (i.e. reliability coefficient ≥ 0.70) during the 7-day monitoring period. WASO required at least 6 nights of monitoring for PLWH and 4 nights for PWoH. Reliable estimates of sleep diary–reported sleep quality were obtained with at least 2 nights of monitoring among PLWH and 3 nights among PWoH.

Table 3 presents ICC estimates restricted to weekday (Monday–Thursday) and weekend (Friday–Sunday) measurements. Among PLWH, within-individual variability in sleep duration was greater on weekdays than on weekends, whereas the opposite pattern was observed for PWoH. Self-reported sleep quality showed much lower within-individual variability in both PLWH and PWoH (both weekdays and weekends).

**Table 3:** Estimates of intraclass correlation coefficients (ICC)^*^ and number of nights of measurement required for the reliable measuring of objective sleep parameters and subjective sleep quality

	Monday through Thursday		Friday through Sunday	
	ICC	Nights Required	ICC	Nights required
Nocturnal sleep duration	0.149^a^ / 0.302^b^	(>7^a^) / (6^b^)	0.286^a^ / 0.207^b^	(6^a^) /(>7^b^)
Sleep latency	0.083^a^ / 0.022^b^	(>7^a^) / (>7^b^)	0. 116^a^ / 0.000^b^	(>7^a^) /(>7^b^)
Wake after sleep onset	0. 330^a^ / 0. 307^b^	(5^a^) / (6^b^)	0. 244^a^ / 0. 447^b^	(>7^a^)/ (3^b^)
Self-report sleep quality^*^	0. 606^a^ / 0. 478^b^	2^a^ / 3^b^	0. 674^a^ / 0. 549^b^	2^a^/>2^b^

^*^The ICC quantifies how much of the variability in a single night of measurement reflects stable differences between participants rather than day-to-day variability within participants.

^a^PLWH.

^b^PWoH.

1. Self-reported sleep quality from sleep diary. Results from when only weekdays are included in the period of measurement (Monday through Thursday) and from when only weekend days are included (Friday through Sunday). When 3 (Friday–Sunday) or 4 (Monday–Thursday) nights were not sufficient to reach reliability (Spearman-Brown coefficient > 0.70), number of nights required are presented in parentheses.

## Discussion

This study extends our previous population-level characterization of sleep health in the Mwanza HIV & CVD Cohort by examining sleep over seven consecutive nights using wrist actigraphy and daily sleep diaries. Whereas our earlier study identified modest differences in selected subjective and objective sleep parameters between PLWH and PWoH based on a single-night assessment, the present study demonstrates that these differences are accompanied by greater within-person variability in sleep duration among PLWH and provides empirical guidance on the number of monitoring nights required for reliable sleep assessment. Specifically, we found that PLWH had shorter sleep duration and greater night-to-night variability in sleep duration than PWoH, despite broadly similar sleep timing, sleep hygiene, and self-reported sleep quality. Across both groups, actigraphy-derived sleep measures exhibited considerable within-person variability, requiring up to seven nights of monitoring to obtain reliable estimates. These findings complement our earlier work by showing that differences in sleep health between PLWH and PWoH extend beyond single day measurements to include sleep regularity and measurement reliability, underscoring the importance of multi-night objective sleep assessment in SSA.

In our study, PLWH had a mean sleep duration of 364 minutes (approximately 6.1 hours per night), which falls within the range reported in prior actigraphy-based studies. For example, studies by Balthazar et al. [33] and Esquivel-Mendoza et al. [34], conducted in the United States (Northeast Ohio and Miami, Florida, respectively), have reported average sleep durations of 6 to 6.5 hours among PLWH using wrist actigraphy, indicating relatively short sleep duration in this population.

Compared with PWoH, PLWH in our study exhibited a modest but statistically significant reduction in sleep duration, with an adjusted mean difference of 32 minutes per night. Although modest in absolute terms, this reduction is consistent with previous actigraphy-based studies of PLWH and contributes to an overall sleep duration of approximately 6.1 hours per night, below the recommended duration for healthy adults. The observed shorter mean sleep duration in PLWH appears to be driven by the cumulative effects of a slightly delayed sleep onset, earlier morning awakening, and increased WASO compared with PWoH. Although none of these differences in individual parameters were statistically significant, their combined effect results in a reduction in total sleep duration for PLWH in our study. This pattern aligns with findings from the study by Redman et al. [35], conducted in a rural South African cohort, who similarly reported shorter sleep duration among PLWH characterized by delayed sleep onset and earlier sleep offset. These findings suggest that HIV-associated sleep disturbance in SSA may manifest as a reduction in total sleep duration driven by alterations in sleep–wake patterns.

Complementing the findings on mean sleep duration, an analysis of sleep regularity demonstrated that PLWH experienced greater night-to-night variability in sleep duration than PWoH, suggesting less regular sleep patterns. This finding is consistent with the actigraphy-based POPPY cohort, which reported greater night-to-night variability in sleep duration and other sleep parameters among PLWH compared with PWoH in the United Kingdom and Ireland [36]. It is interesting to note that the SHI scores in our study were both low and comparable between groups (23.5 for PLWH vs. 24.0 for PWoH), indicating generally favorable sleep hygiene practices. Similarly, self-reported sleep quality was relatively good across groups (15.6 for PLWH vs. 15.8 for PWoH). Collectively, these findings suggest that differences in night-to-night sleep variability are unlikely to be primarily explained by overt maladaptive sleep behaviors. Rather, they may reflect underlying physiological and psychosocial factors associated with living with HIV or ART side effects, although these were not directly measured in the present study. Although the observed between-group difference in sleep duration variability was modest (approximately 13 minutes), it represents a consistent increase in within-person fluctuation in sleep duration among PLWH. At present, clinically meaningful thresholds for night-to-night sleep variability have not been established, making it difficult to determine the direct clinical implications of a difference of this magnitude. Nevertheless, the finding indicates less stable habitual sleep patterns among PLWH and provides additional evidence that HIV-related differences in sleep extend beyond average sleep duration alone.

When considered alongside our previous findings from the same cohort, these results provide additional context. In that study, sleep apnea prevalence was similar between PLWH and PWoH, while excessive daytime sleepiness was less common among PLWH [22]. These findings suggest a discordance between objective indicators of sleep disruption and subjective daytime experiences. This pattern raises the possibility that psychosocial adaptation, health system engagement, or differences in symptom perception may influence how sleep disturbances manifest and are reported among PLWH in SSA.

Sleep regularity is increasingly recognized as a key dimension of sleep health with long-term implications for morbidity and mortality that are independent of sleep duration [14, 37]. Early work by Buysse and colleagues demonstrated that night-to-night variability was associated with impaired daytime functioning [3]. More recent epidemiological research has linked irregular sleep patterns to adverse cardiometabolic outcomes including hypertension and metabolic dysfunction [2]. Rather than relying on established clinical thresholds, current evidence conceptualizes sleep regularity as a continuous dimension of sleep health, with increasing sleep irregularity consistently associated with poorer health outcomes [15]. Accordingly, although the absolute difference observed in our study was modest, it is consistent with an emerging body of evidence suggesting that reduced sleep regularity may represent an important marker of cardiometabolic risk. Whether differences of this magnitude translate into clinically meaningful health consequences remains uncertain and warrants investigation in longitudinal studies. From a population health perspective, even modest shifts in sleep regularity may be important if confirmed to predict adverse outcomes, particularly among PLWH who already experience an elevated burden of cardiometabolic disease [38].

Overall, our results show that three additional nights of measurement are required for reliable actigraphy-derived sleep measures compared with self-reported sleep quality. This observation was expected, as actigraphy-derived sleep parameters exhibit night-to-night variability and are sensitive to short-term behavioral and environmental influences [32, 39]. Consequently, multiple nights of measurement are required to average this within-person variability and obtain reliable estimates [40]. In contrast, self-reported sleep quality reflects a broader subjective perception that tends to remain more stable across nights.

In this study, actigraphy-derived sleep duration for both PLWH and PWoH required 7 nights of monitoring to achieve the desired reliability coefficient (i.e. 0.70). For WASO, PLWH and PWoH needed 6 and 4 nights, respectively. These findings are similar to those reported in other studies. For example, Aili and colleagues, in a study of personnel at a correctional institution, reported that seven or more days of measurement were required to obtain reliable estimates when individuals who worked on weekends were excluded [40]. A recent large study of 10 412 participants reported that reliable intraindividual means for sleep duration and WASO were achieved in 7 and 5 days, respectively [41]. The close agreement between our findings and those of previous studies suggests that the number of monitoring nights required to estimate habitual sleep reliably may be relatively robust across diverse populations and settings, despite differences in geography, lifestyle, and HIV prevalence. Importantly, our study extends this evidence to SSA, where objective sleep research remains limited. Our data demonstrate that the recommended practice of collecting approximately one week of actigraphy data is also appropriate in this setting. These findings provide empirical support for future epidemiological studies and intervention trials in SSA by indicating that seven consecutive nights of monitoring are likely sufficient to obtain reliable estimates of commonly reported actigraphy-derived sleep measures.

Our reliability analyses focused on habitual (mean) sleep measures, as these represent the primary outcomes reported in most epidemiological and clinical actigraphy studies. In contrast, determining the monitoring duration required to obtain reliable estimates of sleep variability metrics is a distinct methodological question that was beyond the scope of the present study. Future studies should compare seven-day and longer monitoring protocols in this population to determine the duration required to obtain reliable estimates of sleep variability metrics.

This study has several strengths. Sleep health parameters were assessed using objective actigraphy over multiple consecutive nights in a natural condition in a well characterized population. The inclusion of both PLWH and PWoH enabled direct comparison within the same community context. The use of sleep diaries alongside actigraphy also provided complementary information, helping accurately determine bedtime and wake-up time. However, several limitations should be considered. The relatively small sample size may limit the precision of some estimates and reduce the ability to detect smaller differences between groups. The study participants of the Mwanza HIV&CVD Cohort are representative of PLWH in urban Tanzania with well-controlled disease on ART, which may limit generalizability to other populations. In addition, PWoH were recruited from a pool of treatment partners attending the same clinic and therefore may not represent a random sample of the general population without HIV. Another limitation is the actigraph’s inability to definitively distinguish between prolonged sedentary bouts and device non-wear time, potentially leading to an overestimation of inactive behavior. To mitigate this, we excluded non-wear intervals by identifying periods characterized by a precipitous drop in ambient temperature and the absence of life-measure signals. In addition, declines in ambient light intensity were used to help identify lights-off events and refine estimation of the intended sleep period.

In conclusion, PLWH demonstrated shorter and less consistent sleep than PWoH, indicating reduced sleep consolidation despite preserved circadian timing. These disturbances, together with the within-person night-to-night variability observed in actigraphy, underscore the importance of multi-night objective assessment when characterizing sleep in this population. The divergence between self-reported sleep ratings and actigraphy-derived parameters further suggests a need for community-level education on sleep health in SSA, particularly regarding sleep regularity. Given that poor sleep health is common among PLWH and may contribute to accelerated cardiometabolic aging, integrating routine sleep screening into HIV clinical care could facilitate earlier identification of at-risk individuals. Finally, clinical trials are needed to elucidate causal pathways and develop targeted interventions to improve sleep and mitigate downstream cardiometabolic consequences in PLWH.

## Data Availability

The data underlying this article will be shared on reasonable request to the corresponding author.
